# Green Routes: Exploring Protein-Based Virus-like Nanoparticle Transport and Immune Activation in *Nicotiana benthamiana* for Biotechnological Applications

**DOI:** 10.3390/vaccines12080831

**Published:** 2024-07-23

**Authors:** Romano Josi, Alessandro Pardini, Alexander Haindrich, Sanjana V. Marar, Anne-Cathrine S. Vogt, Arthur Gessler, Doris Rentsch, Paolo Cherubini, Martin F. Bachmann, Mona O. Mohsen

**Affiliations:** 1 Department for BioMedical Research, University of Bern, 3008 Bern, Switzerlandmona.mohsen@unibe.ch (M.O.M.); 2 Department of Rheumatology and Immunology, University Hospital of Bern, 3010 Bern, Switzerland; 3 Graduate School for Cellular and Biomedical Sciences (GCB), 3012 Bern, Switzerland; 4 Institute of Plant Sciences, University of Bern, 3013 Bern, Switzerland; 5 WSL, Swiss Federal Institute for Forest Snow and Landscape Research, 8903 Birmensdorf, Switzerland; 6 Institute of Terrestrial Ecosystems, ETH Zürich, 8092 Zürich, Switzerland; 7 Deptartment of Forest and Conservation Sciences, University of British Columbia, Vancouver, BC V6T 1Z2, Canada; 8 Tajarub Research & Development, Doha P.O. Box 12627, Qatar

**Keywords:** virus-like particle, nanoparticles, tobacco plant, *Nicotiana benthamiana*, plant defense

## Abstract

Viral, bacterial, fungal, and nematode infections cause significant agricultural losses, with limited treatment options, necessitating novel approaches to enhance plant defense systems and protection against pathogens. Virus-like nanoparticles (VLPs), extensively used in animal and human therapies (e.g., vaccines and immune enhancers), hold potential for novel agricultural solutions and advancing plant nanotechnology. This study employed various methodologies, including VLP production, confocal microscopy, and real-time qPCR. Our findings demonstrated the presence of 30 nm Qβ-VLPs, fluorescently labeled, within the intercellular space of *Nicotiana benthamiana* leaves one hour post-infiltration. Furthermore, infiltration with Qβ-VLPs led to an upregulation of key defense genes (NbPR1a, NbPR5, NbNPR, NbERF1, NbMYC2, and NbLRR2) in treated plants. Using RT-qPCR, a significant increase in the relative expression levels of defense genes was observed, with sustained high levels of NbERF1 and NbLRR2 even after 24 h. These findings suggest that Qβ-VLPs effectively upregulate genes crucial for pathogen defense in *N. benthamiana*, initiating PAMP-triggered immunity and launching signaling cascades that enhance defense mechanisms. This innovative application of VLPs to activate plant defense programs advances plant nanobiotechnology, offering new agricultural solutions.

## 1. Introduction

Plants serve as the cornerstone of human and livestock nutrition. Among plants, there is competition for water, nutrients, and light. Additionally, their development, growth, and health are constantly challenged by various pathogens such as viruses, fungi, bacteria, and nematodes, especially when cultivated in extensive monocultures or controlled environments like greenhouses, where the spread of diseases can be more problematic [1]. Viral diseases, such as Cucumber Mosaic Virus (CMV), Tobacco Mosaic Virus (TMV), Tomato Mosaic Virus (ToMV), and Eggplant Mosaic Virus (EMV), account for around 10–15% of total crop infections globally, resulting in annual economic losses amounting to billions of dollars [2,3]. Fungal infections, including powdery mildew caused by fungi such as Erysiphales, are particularly significant, accounting for approximately 10–23% of crop infections. Bacterial infections caused by pathogens like *Xanthomonas* and *Pseudomonas* species are responsible for about 10–15% of crop damage. Nematode infections, particularly from species like *Meloidogyne* spp., contribute to around 12% of crop damage by impairing root systems, which disrupts water and nutrient absorption [2,3,4,5].

Herbicides provide an effective solution for controlling weeds, while fungicides are employed to combat fungal infections. Non-specific disinfectants are commonly used to address both fungal and bacterial infections. Despite viruses accounting for significant crop loss in agriculture, treatment options for controlling this class of pathogens remain limited [6,7]. 

When plants are infected by pathogens, the activation of their defense responses is regulated. The first line of defense is the recognition of pathogen-associated molecular patterns (PAMPs) by cell-surface localized pattern-recognition receptors (PRRs) [8]. Afterward, the plant induces PRR-triggered immunity such as calcium ions (Ca^2+^), reactive oxygen species (ROS), and mitogen-activated protein kinase (MAPK) activation. The JA/ET-related defense pathway will be activated in a next step, which is mediated by two key signaling molecules: the plant hormones jasmonic acid (JA) and ethylene (ET). This process leads to the biosynthesis of defense-related factors and disease resistance in the plant [8]. Numerous genes associated with pathogen-related stress and defense mechanisms have been discovered and extensively investigated in plants. They can be categorized into (a) pathogen-related genes (e.g., *NbPR1a* and *NbPR5*), which play a crucial role in systemic acquired resistance by enhancing the plant’s defense against a diverse array of pathogens [9], (b) defense-related regulatory genes (e.g., *NbNPR1* and *NbERF1*), which upregulate the expression of other defense-related genes, coordinate complex signaling pathways to activate defense mechanisms, and, finally, (c) signaling and receptor genes (e.g., *NbMYC2* and *NbLRR2*) [10,11]. 

Virus-like nanoparticles (VLPs) are complex supramolecular structures composed of multiple proteins, possessing various virus-like features that can be strategically utilized in viral nanotechnology [12]. VLPs are regarded as pathogen-associated structural patterns (PASPs) that can initiate both innate and adaptive immune responses in humans and animal models [13,14,15]. VLP-based immunotherapy shows key virus characteristics while eliminating the potential for replication, as they lack a viral genome. This absence of genetic material makes them a secure platform for various biotechnological applications [16]. VLPs can be synthesized in over 170 diverse expression host systems, encompassing bacteria, insects, yeast, or mammalian cells, reflecting in part the broad host spectrum of the viruses, which VLPs are derived from [17]. Approximately 30% of VLPs are produced using bacterial systems, with *E. coli* being the primary host organism. This method offers the advantage of very high yield and low production costs, enabling the efficient manufacturing of large amounts of VLPs, in particular, bacteriophage-derived VLPs [18], potentially also for use in agriculture. The genes encoding viral structural proteins are codon optimized for bacterial expression and inserted into commercial plasmids under robust promoters allowing the efficient production of the intended recombinant proteins in *E. coli* [19,20]. The field of bacteriophages, often referred to as phages, which prey on bacteria and archaea, represents a significant portion of the vast universe of viruses. Undoubtedly, phages are the most abundant organisms on Earth, with their numbers exceeding 10^31^ units, making them the most remarkable preservers of genetic diversity in the Earth’s biosphere. The immense impact of phages across diverse fields of life sciences mirrors their remarkable abundance. Among them are the single-stranded positive-sense RNA phages of the *Leviviridae family* [21]. Charles Weissmann’s team predicted in the 1970s that gene engineering would become dominant in RNA phage studies [22,23]. In their paper on site-directed mutagenesis, the authors recognized that expressing altered DNA in vivo or in vitro may facilitate the creation of proteins with specific amino acid substitutions. At the same time, the bacteriophage Qβ RNA was carefully prepared for use in cloning and expression techniques [24]. Qβ virus-like particles (Qβ-VLPs) are non-infectious, protein-based nanoparticles approximately 30 nm in size, mimicking the structure of the bacteriophage Qβ virus [25]. These recombinant Qβ-VLPs consist of 180 capsid proteins (CPs) that self-assemble into *T* = 3 icosahedral particles. Importantly, Qβ-VLPs lack genetic material, making them safe platforms for the development of prophylactic and therapeutic vaccines, as well as immune activators and enhancers [13].

There is a lack of information on whether protein-based nanoparticles such as Qβ-VLPs can enter plant leaves and activate the plant’s immune defense mechanisms. Protein-based VLPs offer the advantage of being biocompatible and biodegradable, potentially reducing environmental impact. Additionally, using VLPs may result in activating different defense mechanisms in the plant, providing enhanced protection against infections and pathogens. Exploring this aspect could significantly advance the field of VLP-based nanotechnology in plant science.

Here, we demonstrate that protein-based Qβ-VLPs could efficiently be visualized in the intercellular space of *N. benthamiana* leaves upon infiltration and importantly activate several plant-defense systems within an hour after the exposure. Using appropriate technology, this observation may be harnessed at a large scale by rapidly rendering plants resistant to infection, in particular viral infection.

## 2. Materials and Methods

### 2.1. Qβ-VLP Purification

Glycerol stock of Qβ transformed JM109 cells (obtained from Bachmann Lab, JM109 cells are commercially available) were transferred to LB medium (1% (*w*/*v*) tryptone, 0.5% (*w*/*v*) yeast extract, 1% (*w*/*v*) NaCl) containing 100 µg/mL ampicillin. Bacteria were incubated overnight at 37 °C. Then, 10 mL of overnight culture was inoculated in M9 medium for 18 h at 37 °C under agitation. Cells were then centrifugated at 4000× *g* for 30 min. The pellet was resuspended in lysis buffer (20 mM NaPO_4_, 1% Triton X-100, 100 mM EDTA, Benzonase, Lysozyme, Protease inhibitor, pH 7.5) and incubated at room temperature for 90 min. Lysed cells were sonicated for 3 min. The lysate was centrifuged for 25 min (4 °C at 14,000× *g*). The supernatant was collected and filtered (0.2 μm). The lysate was subsequently concentrated to obtain around 10 mL of Qβ. Using a FRACTOGEL column for anion exchange, Qβ was further purified. Running buffer A (20 mM NaPO_4_ pH 7.2, 150 mM NaCl) and elution buffer B (20 mM NaPO_4_ pH 7.2, 1 M NaCl) were used. Elution was performed by a linear gradient starting with 10% of buffer B over 10 column volumes. Fractions were collected by a fraction collector. Collected fractions from the size exclusion column were analyzed by SDS-PAGE, and fractions containing Qβ were concentrated using Amicon 100 kDa columns. Qβ concentration was determined using Nanodrop and BCA (Pierce™ BCA Protein Assay Kit, Thermo Fisher, Basel, Switzerland). The plasmid map and Qβ sequence can be found in Supplementary Figure S1 and Supplementary Table S1.

### 2.2. Electron Microscopy

VLPs were visualized by transmission electron microscopy (Philips CM12 EM) to assess integrity and shape. Sample grids were glow discharged. Afterwards, 10 μL of Qβ-VLPs (1 mg/mL) was added for 30 s. Afterward, grids were washed 3× with ddH_2_0 and negatively stained with 5 μL of 5% uranyl acetate for 30 s. The grids were allowed to air dry for ten minutes after any excess uranyl acetate was pipetted away. For the analysis of the VLPs, 84,000× and 110,000× magnifications were used to capture the images.

### 2.3. Labeling of Qβ-VLPs with Alexa Fluor 488

Qβ-VLPs in a concentration of 2 mg/mL were used for the labeling procedure. The Alexa Fluor 488 NHS Ester Thermo Fisher kit was used to label the VLPs according to the manufacturer’s instructions. Briefly, 1 µL of aliquoted dye was added to 1 mL of 2 mg/mL VLPs. The sample was mixed well and incubated for 1 h on a thermal shaker at room temperature. To wash away excessive AF488, Zebra spin desalting columns with a 7 kD MWCO (Thermo Fisher, 89882) were used. 

### 2.4. Nicotiana benthamiana

Soil-grown *N. benthamiana* plants were cultivated in a growth chamber under long-day conditions (14 h light/10 h dark, 140 µmol m^−2^ s^−1^, at 24 and 18 °C). Leaves of 5–6-week-old plants were used for the experiments. 

### 2.5. Confocal Microscopy

*Nicotiana benthamiana* plants were exposed to higher light (220–240 µmol m^−2^ s^−1^) for 30 min to induce full opening of stomata. Thereafter, leaves were infiltrated with 1 mL labeled Qβ-VLP solution (200 μg/mL) by using a syringe and incubated for one hour before visualization by confocal microscopy. The infiltration caused visible wet spots, which were marked to ensure that non-wet leaf tissue could also be imaged. For imaging, the leaves were cut into smaller pieces (ca. 2 × 1 cm^2^), which were mounted on a glass slide with cover glass. Samples were visualized using a Leica SP5 Confocal Microscope. An ×40 objective was used with an excitation laser at 488 nm, and emission was collected at 500–550 nm for Alexa488 and 650–725 nm for autofluorescence of chloroplasts. For each picture, around 40 Z-stacks were taken in a step size of 1.01 μm. Images were analyzed by ImageJ software (Version 1.54i), and 3D reconstruction of the Z-stack was performed using Imaris imaging software. 

### 2.6. RNA Isolation from N. benthamiana Leaves

Snap-frozen leaves were put in Eppendorf tubes and homogenized using a pistil and mechanical force. Trizol reagent (Thermo Fisher, 15596026) was added to the Eppendorf tubes, vortexed for 30 s, and incubated on ice for 10 min. The tubes were centrifuged for 10 min at 12,000× *g* and 4 °C. The supernatant was transferred into new tubes and the pellet was discarded. Ice-cold chloroform was added to each tube, vortexed, and incubated on ice for 10 min. The tubes were centrifuged for 15 min at 12,000× *g* and 4 °C. The upper phase of the supernatant was transferred into fresh tubes. Ice-cold isopropanol was added and mixed by inverting. The samples were incubated for 10 min on ice and subsequently centrifuged for 10 min at 12,000× *g* and 4 °C. The supernatant was discarded, 75% ethanol was added to the pellet, and the tube was gently vortexed. The samples were centrifuged for 5 min at 8000× *g* at 4 °C. The supernatant was discarded, and the pellet was dried on a heat block for 10 min at 24 °C. The pellet was resuspended in 50 µL of nuclease-free water (Thermo Fisher, AM9937). The RNA content was measured using Nanodrop (Witec AG, Sursee, Switzerland). Then, cDNA was produced using the high-capacity RNA-to-cDNA kit (Thermo Fisher, 4387406) according to the manufacturer’s instructions, and cDNA content was again measured using Nanodrop.

### 2.7. Quantitative Real-Time PCR

Quantitative real-time PCR (RT-qPCR) was used to examine the relative transcript levels of different genes. Here, we used *NbGAPDH* as the reference gene, and the gene-specific primers that we used are listed in Table 1. SYBR™ Select Master Mix (Thermo Fisher) and the QuantStudio™ 6 Flex (Thermo Fisher) were used to perform these experiments. Then, 2 μL of cDNA, 10 μL SYBR™ Select Master Mix, 1 μL of each forward and reverse gene-specific primer (10 μM), and 6 μL ddH_2_O were added to the 20 μL reaction mixtures. Amplification of the cDNA was performed as follows: 95 °C for 10 min, then 50 cycles of denaturation at 95 °C for 15 s, and then 60 °C for 60 s. In the end, melting curve analyses were included. For the evaluation of the data, we used Design & Analysis 2 (DA2, Thermo Fisher) with the $2^{-\Delta \Delta Ct}$ method with NbGAPDH as reference gene [26]. The expression level of the genes was calculated from three technical replicates from both Qβ-VLP-treated and PBS 1x-treated plants after 1 hpi and 24 hpi.

### 2.8. Statistical Analysis

The data were analyzed and presented as mean ± SEM using GraphPad V.10.2.0 (464). Statistical comparisons between two groups utilized Student’s *t*-test. Significance levels were denoted as **** *p* < 0.0001, *** *p* < 0.001, ** *p* < 0.01, and * *p* < 0.05.

## 3. Results

### 3.1. Expression, Production, and Labeling of Protein-Based Bacteriophage Nanoparticles Qβ-VLPs

**Figure 1:**
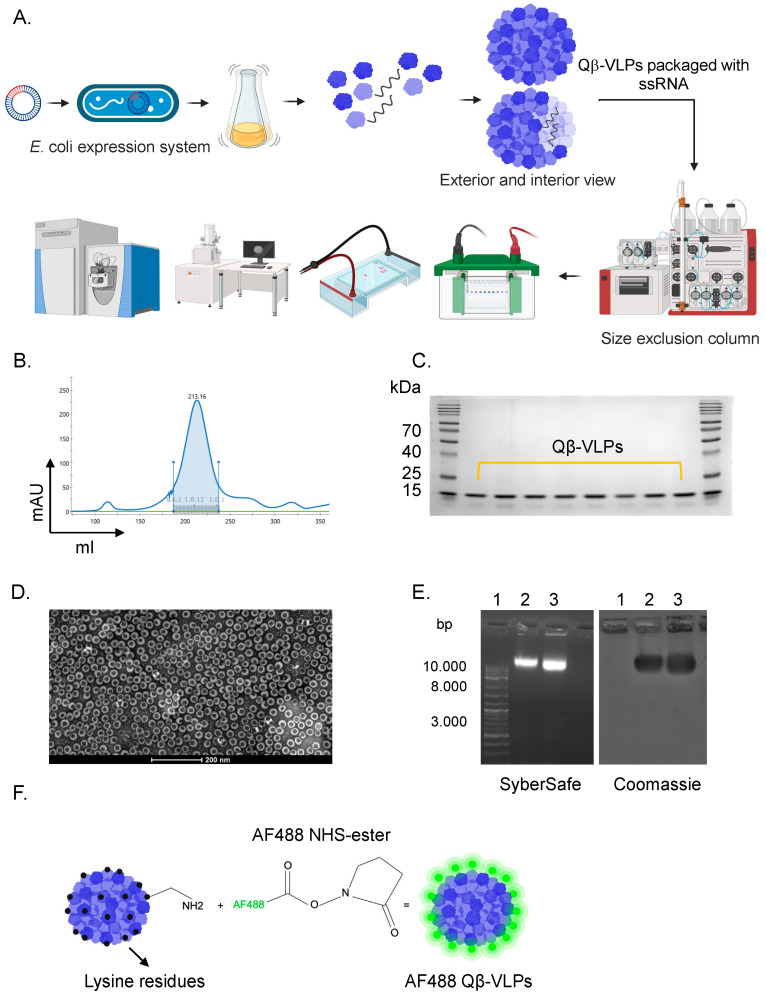
(**A**) An illustration showing the production procedure of Qβ-VLPs. Qβ-VLPs were expressed in *E. coli*, purified with a size exclusion column, and verified for their shape and integrity. (**B**) Sephacryl S500 HR size exclusion peak of Qβ-VLPs. Fractions were collected starting at 30 mAU. (**C**) A 12% SDS-PAGE gel showing fractions of Qβ-VLPs after sephacryl S500 HR size exclusion. The signal at 15 kDa indicated the subunits of Qβ-VLP. (**D**) Electron microscopy image of a pooled fraction of Qβ-VLP. (**E**) The 1% agarose gel analysis of Qβ-VLP containing nucleic acid within the particle. 1. DNA ladder, 2. Qβ-VLP, 3. Qβ-AF488. The gel was imaged once with SyberSafe on the left and once with Coomassie Blue on the right. (**F**) An illustration showing the fluorescent labeling of Qβ-VLP with AlexaF488 via lysine residues.

Protein-based nanoparticles derived from the bacteriophage Qβ-VLPs are easily produced at large scale, as depicted in Figure 1A. The recombinant Qβ-VLPs are composed of 180 subunits or CPs, which autonomously assemble into *T* = 3 icosahedral particles closely resembling the parental virus [28]. Each subunit or monomer has an approximate molecular mass of 14 kDa and they are arranged in pentamers and hexamers. In contrast to replicating viruses, Qβ-VLPs do not contain replication-competent viral genetic material and no infectious potential, making them inherently safe for applications in nanobiotechnology. Qβ-VLPs can be effectively expressed in bacteria, such as *E. coli*. In our study, JM109 *E*. *coli* was utilized for expression, followed by purification using a combination of fractogel and size exclusion columns, as illustrated in Figure 1B. To confirm the successful expression of Qβ-VLPs and the integrity of the particles, we performed SDS-PAGE, electron microscopy, and agarose gel analysis. The SDS-PAGE showed a protein at ~14 kDa, which is equivalent to a monomer (subunit) of Qβ-VLPs (Figure 1C). Electron microscopy showed nicely formed particles of ~30 nm (Figure 1D). During the expression process in *E*. *coli*, Qβ-VLPs naturally package ssRNA from the bacterial host, as shown in Figure 1E. The packaging of the negatively charged ssRNA imparts an overall negative charge to the nanoparticles, as evidenced by their migration pattern in the agarose gel. These data confirm the successful expression and production of uniform icosahedral Qβ-VLPs. Following confirmation of the successful expression and purification of the nanoparticles, we fluorescently labeled Qβ-VLPs with AF488 for imaging and visualization using confocal microscopy. Briefly, AF488 NHS-ester was added to Qβ-VLPs causing the NHS-ester to react with the lysine residues on the VLPs, forming a stable covalent bond between the dye and the VLPs. After the labeling process, purification steps were followed to remove any unreacted dye molecules. An illustration of Qβ-VLPs’ labeling is shown in Figure 1F.

### 3.2. Visualization of Qβ-VLPs in Tobacco Leaves Demonstrates Widespread Distribution

**Figure 2:**
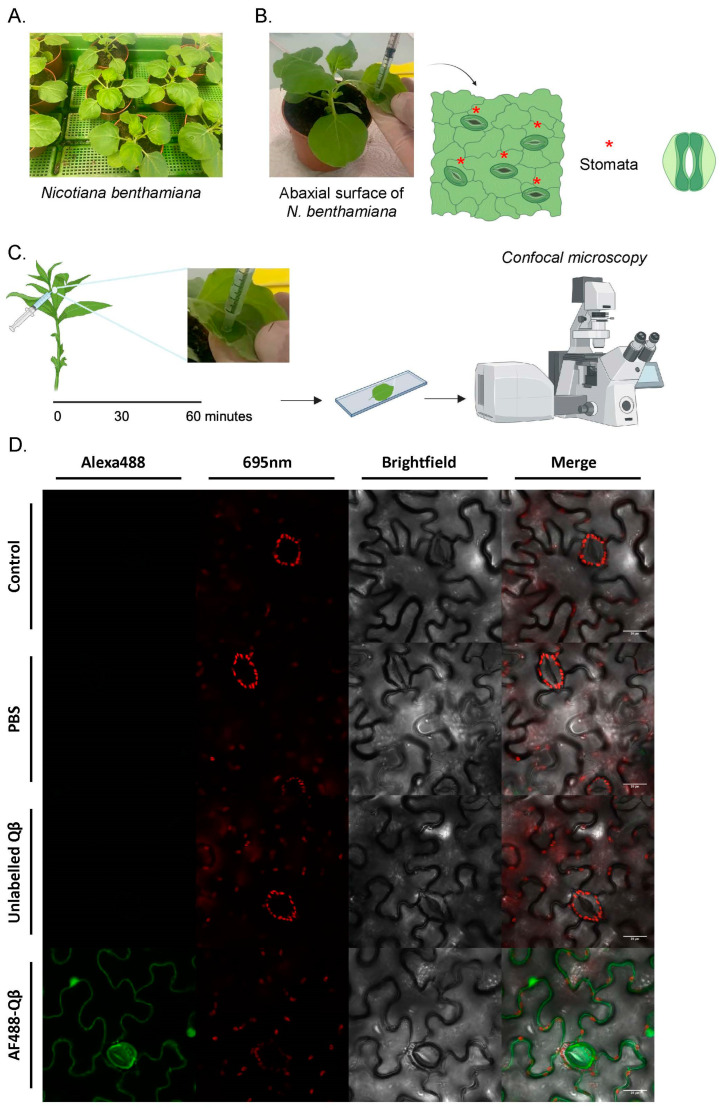
(**A**) *N. benthamiana* plants were cultivated under controlled conditions. (**B**) An image showing the infiltration of Qβ-VLPs on the abaxial surface of the tobacco leaf. The plants were kept under higher light for 30 min before infiltration, forcing the stomata to fully open. Stomata are indicated with a red star. (**C**) Sixty minutes after the infiltration of the different solutions, a leaf section of a wet or non-wet spot was excised and analyzed under the confocal microscope. (**D**) Representative images showing leaf sections of different treatments: untreated control, infiltrated with PBS, infiltrated with unlabeled Qβ-VLP, and infiltrated with AF488-Qβ. In the red channel at 650–725 nm, chloroplast autofluorescence is detected. Scale bars at 20 µm.

To explore the visibility of our protein-based nanoparticles within plants, we chose *Nicotiana benthamiana,* a *Nicotiana* species closely related to common tobacco (*Nicotiana tabacum*), as our model system. This choice was due to its widespread use in plant research, its suitability for experimental manipulation and viral infection, and its established role as a model organism in plant biology (Figure 2A). We utilized confocal microscopy to achieve high-resolution visualization of the fluorescently labeled nanoparticles in the tobacco leaves. Accordingly, we applied our AF488 Qβ-VLPs to the abaxial surface of the tobacco leaf through infiltration, referred to here as infiltration (Figure 2B). This method involves gently administering the virus-like particles (VLPs) to the leaf surface without applying force, ensuring that the nanoparticles infiltrate the leaf tissue smoothly and uniformly. Various controls were included, such as leaves that were not infiltrated as a baseline, leaves infiltrated with PBS, and unlabeled Qβ-VLPs. The PBS control is crucial to reveal any potential confounding effects of PBS on the tobacco leaves. The tobacco leaf, infiltrated with the specified solutions, underwent examination through confocal microscopy one hour post-infiltration (Figure 2C). We evaluated the infiltrated leaves employing confocal microscopy through two distinct fluorescence emissions: the 650–725 nm emission for chlorophyll autofluorescence and 500–550 nm for Alexa Fluor 488. Our results confirmed the presence of 30 nm AF488 Qβ-VLPs in the plant leaves one hour following the infiltration, as shown in Figure 2D. Since the stomata exhibited fluorescence, we speculated that the AF488 Qβ-VLPs adhered to the plant cell wall following their administration. Confocal microscopy Z-stack projections were generated for each experimental group, providing the 3D distribution of the AF488 Qβ-VLPs following infiltration (Supplementary Videos S1–S5).

**Figure 3:**
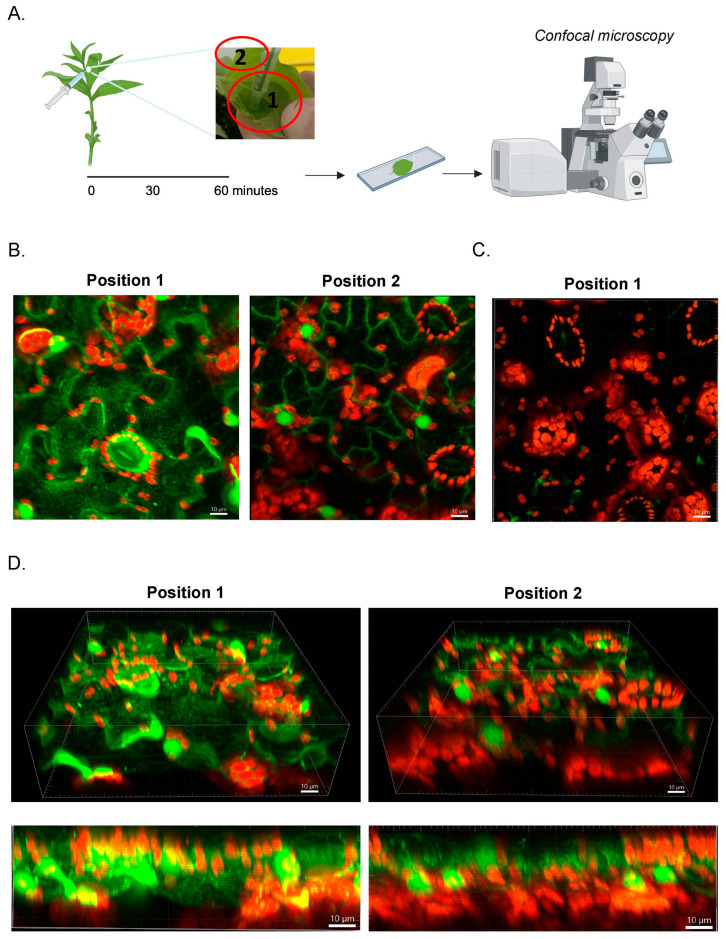
(**A**) The two sites of analysis are shown. Infiltration of Qβ-AF488, Qβ-VLP, and PBS was performed as shown in the picture. Position 1, the location of infiltration termed “wet”, and position 2, further away from the infiltration site, termed “non-wet”. From both positions, leaf sections were excised and analyzed with a confocal microscope. (**B**) Representative Z-projections of Qβ-VLP-infiltrated tobacco leaf from positions 1 and 2. Scale bar at 10 µm. (**C**) Representative image of PBS-infused tobacco leaf from position 1. (**D**) Representative images of Qβ-VLP-infiltrated leaves in side view showcasing the Z-stacks for positions 1 and 2. Scale bars at 10 µm.

Next, we examined how AF488 Qβ-VLPs spread within the leaf tissue following infiltration. This could be promising for potential targeted delivery applications using nanoparticles. AF488 Qβ-VLPs were gently administered to the abaxial leaf surface where a wet spot was formed and labeled as position 1. After one hour, we visualized the diffusion of the labeled nanoparticles from position A to a further dry point on the leaf (position 2) (Figure 3A). Positions 1 and 2 were examined by confocal microscopy as described previously. Our findings showed that AF488 Qβ-VLPs efficiently diffused from position 1 to position 2 and could be visualized after one hour, with significantly higher fluorescence observed at Position 1, where the infiltration took place (Figure 3B). As a control, PBS infiltration was used (Figure 3C). The thickness of *N. benthamiana* leaf ranges between 0.1 to 0.2 mm, which can vary depending on factors such as the age of the leaf, environmental conditions, and specific growing conditions. Therefore, we assessed whether AF488 Qβ-VLPs can be detected beyond the surface of the leaf. We utilized confocal microscopy to generate a stacked projection comprising images from the first abaxial layers of the leaf (40 Z-stacks). The fluorescent signal of AF488 Qβ-VLPs was visible in the intercellular region across all Z-stacks, with a stronger signal observed at position 1 compared to position 2 (Figure 3D and Supplementary Videos S4 and S5). This confirms that AF488 Qβ-VLPs can penetrate and be visualized within the deeper layers of the leaf tissue. However, additional experiments to analyze the exact depth of the VLPs’ penetration would be needed in the future.

### 3.3. Induction of Defense Gene Expression by Protein-Based Nanoparticles in N. benthamiana

**Figure 4:**
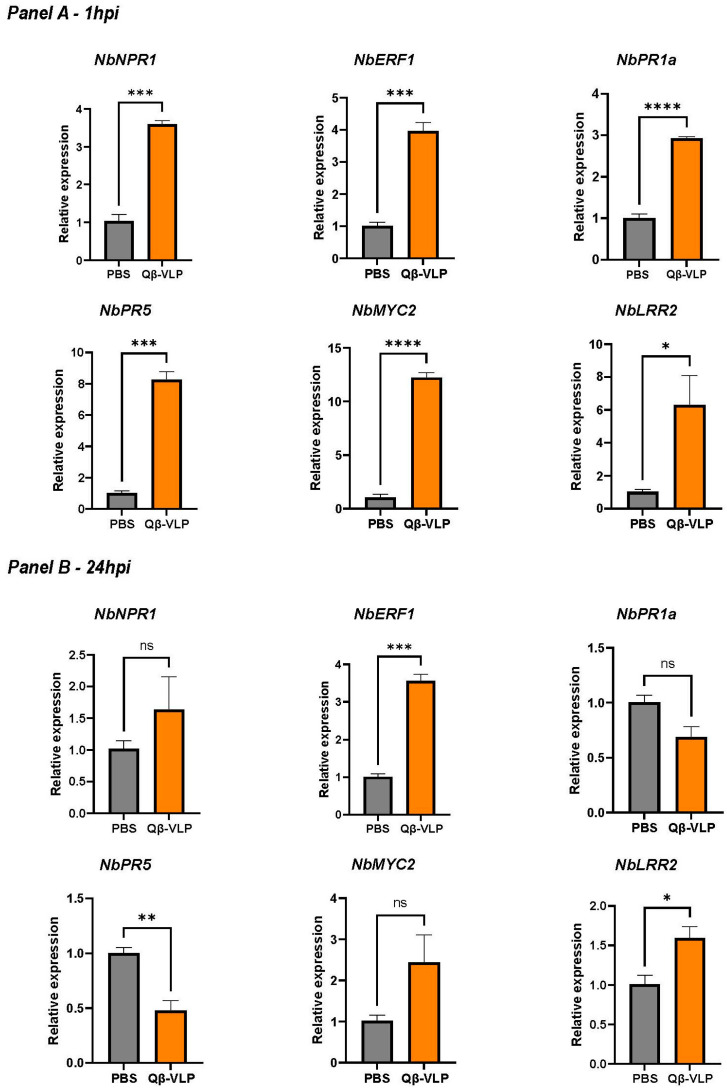
Panel (**A**), relative expression of the six genes of interest: *NbPR1*, *NbERF1*, *NbPR1a*, *NbPR5*, *NbMYC2*, *and NbLRR2*, 1 h post-infiltration (1 hpi). Panel (**B**), relative expression of the same six genes 24 h post-infiltration (24 hpi). Relative expression levels were calculated using the $2^{-\Delta \Delta Ct}$ method, with *NbGAPDH* as the reference gene. The error bars indicate mean ± SEM. Statistical comparisons between two groups utilized Student’s *t*-test. Significance levels were denoted as **** *p* < 0.0001, *** *p* < 0.001, ** *p* < 0.01, and * *p* < 0.05.

To assess the potential of our nanoparticles to induce an immune response in *N. benthamiana*, we evaluated the upregulation of key genes responsible for leaf defense against pathogens [10,11]. We focused on (a) pathogen-related genes (*NbPR1a* and *NbPR5*) [9], (b) defense-related regulatory genes (e.g., *NbNPR1* and *NbERF1*), and (c) signaling and receptor genes (*NbMYC2* and *NbLRR2*) [10,11]. Briefly, non-labeled Qβ-VLPs were administered as previously described to the abaxial surface of the leaf. Two experimental groups were formed: one with *N. benthamiana* leaves infiltrated with PBS as a control and the other with leaves infiltrated with AF488 Qβ-VLPs. Leaf samples were harvested at one hour and twenty-four hours post-infiltration to evaluate the expression of the selected genes. Our results showed a significant increase in the mRNA levels of all tested genes one hour following the infiltration of *N. benthamiana* leaves with Qβ-VLPs compared to the control group treated only with PBS. Specifically, there were significant increases in the expression of *NbNPR1* (*p* = 0.0002), *NbERF1* (*p* = 0.0006), *NbPR1a* (*p* < 0.0001), *NbPR5* (*p* = 0.0002), *NbMYC2* (*p* < 0.0001), and *NbLPR2* (*p* = 0.0414) (Figure 4A). Next, we studied the mRNA levels twenty-four hours post-infiltration (Figure 4B). Our results showed a significant, persistent increase in expression, albeit with lower levels compared to the initial spike, in the treated group compared to the control in the defense-related regulatory genes *NbERF1* (*p*. 0.0002) as well as the signaling and receptor genes *NbLRR2* in the group treated with the nanoparticles in comparison to the PBS group (*p* = 0.0335). No significant differences were detected between the treated and control groups in the mRNA levels of *NbNPR1* (*p* = 0.3161), *NbPR1a* (*p* = 0.0517), and *NbMYC2* (*p* = 0.1077) at the twenty-four-hour time point. Interestingly, at the twenty-four-hour time point, mRNA expression levels of *NbPR5* (a pathogen-related protein) were significantly lower in the group treated with Qβ-VLPs compared to the control group (*p* = 0.0072). Taken together, our data indicate that infiltrating *N. benthamiana* leaves with Qβ-VLPs results in the significant upregulation of various genes related to different pathways, including pathogen-related genes, defense-related regulatory genes, and signaling and receptor genes, one hour post-treatment. These findings support our hypothesis that nanoparticles may have potential future applications in agriculture.

## 4. Discussion

In this study, we visualized the uptake of our fluorescently labeled 30 nm protein-based nanoparticles, particularly the bacteriophage Qβ-VLPs, within *N. benthamiana* leaves. We also studied their potential to stimulate an immune defense response in the treated foliage. The goal was to further develop efficient, safe, and biodegradable treatments for different commercially relevant crops such as potatoes, tomatoes, eggplants and other solanceous plants against viruses, bacteria, fungi, and potentially nematodes by exploring the use of protein-based nanoparticles.

Plant cells typically carry a net negative charge on their surface due to the presence of various components, including polysaccharides and proteins within the cell wall. The negative charge on the cell wall is crucial for nutrient uptake, ion exchange, and interactions with other cells or molecules in the environment [29]. Although our Qβ-VLPs incorporated ssRNA during the expression process, contributing to their negative charge, our visualization successfully captured their presence in the intercellular space of the *N. benthamiana* leaves upon infiltration. However, it remains unclear whether the nanoparticles are also localized within the plant cells. We plan to investigate this area in our future research. We also plan to determine whether positively or neutrally charged nanoparticles would exhibit greater efficiency in uptake by plant cells. Altering the net charge of our protein-based nanoparticles can be easily accomplished by digesting the incorporated ssRNA (resulting in neutral VLPs) and by replacing the ssRNA with other molecules such as positively charged ligands (i.e., polyethyleneimine (PEI) or poly-L-lysine), which will result in positive VLPs. Another option will be to conjugate the VLPs with positively charged peptides or polymers.

Napier et al. demonstrated in 2022 an inverse relationship between the uptake of polymer nanoparticles and their size. Specifically, they found that 22 nm negatively charged polymer nanoparticles exhibit a gradual accumulation around roots, ultimately becoming prominently present in the xylem of intact roots over time [30]. In our research, we concentrated on studying the impact of infiltration of protein-based nanoparticles in the leaves of *N. benthamiana*. In our upcoming studies, we aim to study in more depth the potential uptake into cells and long-distance transport of VLPs in plants. This will provide valuable insights into translational applications using the vacuum infiltration methods for plant leaves [31,32] or by adding the nanoparticles directly to the soil.

Our findings showed an efficient diffusion of Qβ-VLPs in the intercellular space of *N. benthamiana* leaves, effectively visualized through confocal microscopy. This can be attributed to the size of the Qβ-VLPs (approximately 30 nm) and the gentle force applied during administration, specifically through infiltration into the abaxial surface of the leaf. Further studies are needed to precisely determine the localization of the VLPs post-administration and potential uptake into cells and intracellular accumulation in different compartments, which will require the additional staining of the cytosol [33], nuclei [34], mitochondria [33], vacuoles [35], as well as the chloroplast [36].

Pathogen-infected tissues generate a mobile immune signal triggering systemic acquired resistance and gene reprogramming. Epigenetic changes and chromatin remodeling help create long-lasting cellular memories and can lead to inherited changes in the genome’s structure [37]. Our results confirmed effective upregulation of expression one hour following treatment with Qβ-VLPs. Enhanced mRNA levels were detected for pathogen-related genes (NbPR1a and NbPR5), which are mostly involved in a PAMP-triggered response [26,38], as well as for defense-related regulatory genes (NbNPR1 and NbERF1) and signaling and receptor genes (NbMYC2 and NbLRR2) [10,11]. The receptors for these pathways are typically located on the cell surface (particularly on the plasma membrane) of leaf cells. Accordingly, it explains the ability of the VLPs to activate these pathways while diffusing in the intercellular space of *N. benthamiana* leaves. *NbMYC2* and *NbLRR2* gene expressions were upregulated one hour after treatment with Qβ-VLPs, and the response continued twenty-four hours later. *NbMYC2* is involved in the jasmonate (JA) signaling pathway, which reacts with the salicylic acid (SA) and ethylene pathways while *NbLRR2* encodes a leucine-rich repeat receptor-like protein involved in recognizing pathogen effectors and initiating defense responses. Salicylic acid (SA) is a critical hormone in plant defense particularly against biotrophic pathogens [39]. Further research to elucidate the exact pathways and mechanisms involved in this response is of high interest to our lab. The decreased expression of *NbPR5* twenty-four hours after injecting the VLPs in the *N. benthamiana* leaf may be explained by having an acute response phase to stress followed by a resituation phase where the response returns to baseline or below (i.e., feedback regulatory mechanisms). Previous studies have demonstrated that localized pathogen attacks in plants can trigger broad-spectrum immunity to reinfection throughout the entire plant and induce immune memory known as systemic acquired resistance. This immune memory can last from a few days to the plant’s entire lifespan and can even be inherited [40]. Therefore, future research should explore the longevity of this induced protection. In summary, our study sheds light on the innovative application of bacteriophage protein-based nanoparticles in advancing plant-based technology and agricultural practices through nanobiotechnology.

## Figures and Tables

**Table 1:** Primers used for real-time PCR.

Gene	Forward Primer (5′–3′)	Reverse Primer (5′–3′)	Reference
**NbPR1a**	GATGCCCATAACACAGCTCG	CGAGGTTACAATCTGCAGCC	[10]
**NbNPR1**	GATACACGGTGCTGCATGTT	AAGCCTAGTGAGCCTCTTGG	[10]
**NbERF1**	GGCGAATTTTCCGGGAGACT	GGCTCCGATTTTACTTCGCC	[10]
**NbMYC2**	GAGATTAGCTGCTTCGCACTG	GCCCGTAGTCGCACCCATA	[10]
**NbLRR2**	TGGAAGGGAAGTAGCAGTG	TACAAGGTTTGGATGAGGC	[10]
**NbPR5**	AACTTCAACGGTGGTGGC	TGAGGGATGGACCGCAAT	[27]
**NbGAPDH**	AGCTCAAGGGAATTCTCGATG	AACCTTAACCATGTCATCTCCC	[11]

## Data Availability

Dataset available on request from the corresponding author.

## Supplementary Materials

The following supporting information can be downloaded at https://www.mdpi.com/article/10.3390/vaccines12080831/s1: Video S1: Buffer Only, Video S2: Control, Untreated, Video S3: VLP No-Lable wet, Video S4: VLP-AF488 Wet, and Video S5: VLP-AF488 no-Wet; Figure S1: pGem–Qβ plasmid, T7 promotor denoted in blue, pTrp in light grey, Sp6 in dark green, Qβ coat protein in green, Qβ Readthrough in grey and ampicillin resistance gene in yellow. Figure was made with Benchling (Biology Software). 2024. Retrieved from https://benchling.com, accessed on 17 July 2024; Table S1: Qβ coat protein sequence.
