# Supplementary material for: Green Routes: Exploring Protein-Based Virus-like Nanoparticle Transport and Immune Activation in Nicotiana benthamiana for Biotechnological Applications
Source: Vaccines (Basel). 2024 Jul 23;12(8):831. doi: 10.3390/vaccines12080831 (PMC11358932; doi:10.3390/vaccines12080831)
Supplement: Supplementary file 1 [file vaccines-12-00831-s001.zip › vaccines-3082884-supplementary figure and table.pdf]

**Supplementary Figure 1**

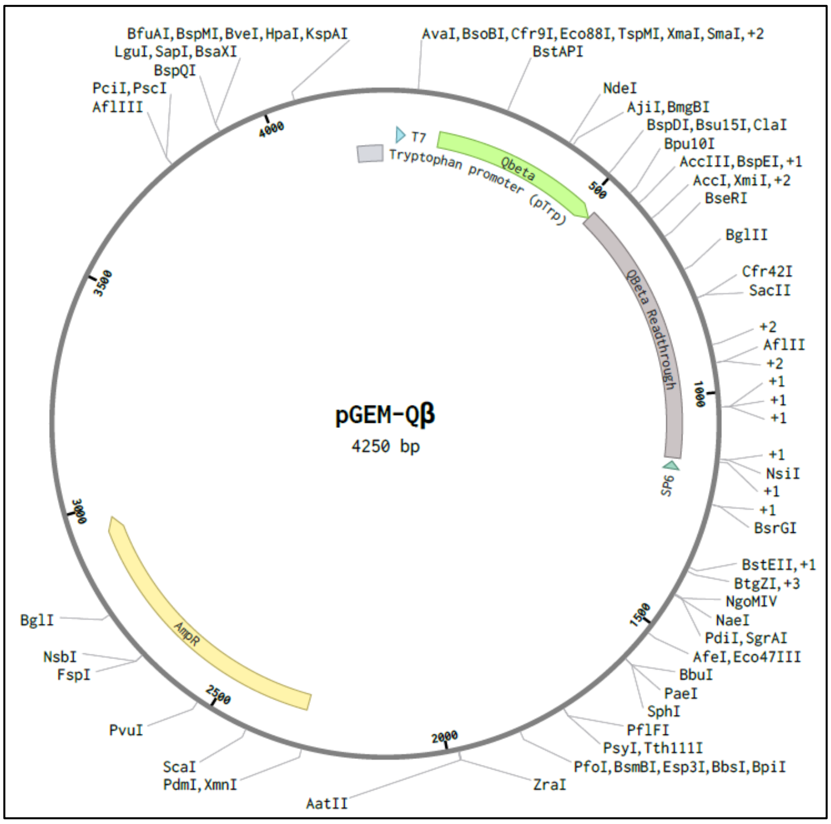


**Supplementary Figure 1.** pGem – Qβ plasmid, T7 promotor denoted in blue, pTrp in light grey, Sp6 in dark green, Qβ coat protein in green, Qβ Readthrough in grey and ampicillin resistance gene in yellow. Figure was made with Benchling (Biology Software). 2024. Retrieved from https://benchling.com.

**Supplementary Table 1. Qβ coat protein sequence**

| Gene | Amino acid sequence |
|---|---|
| **Qβ** | MAKLETVTLGNIGKDGKQTLVLNPRGVNPTNGVASLSQAGAVPALEKR VTVSVSQPSRNRKNYKVQVKIQNPTACTANGSCDPSVTRQAYADVTFSFT QYSTDEERAFVRTELAALLASPLLIDAIDQLNPAY* |
